# Suppression of AGR2 in a TGF-β-induced Smad regulatory pathway mediates epithelial-mesenchymal transition

**DOI:** 10.1186/s12885-017-3537-5

**Published:** 2017-08-15

**Authors:** Lucia Sommerova, Eva Ondrouskova, Borivoj Vojtesek, Roman Hrstka

**Affiliations:** RECAMO, Masaryk Memorial Cancer Institute, Zluty kopec 7, 656 53 Brno, Czech Republic

**Keywords:** AGR2, EMT, TGF-β, E-cadherin, Vimentin, Metastasis

## Abstract

**Background:**

During cancer progression, epithelial cancer cells can be reprogrammed into mesenchymal-like cells with increased migratory potential through the process of epithelial-mesenchymal transition (EMT), representing an essential step of tumor progression towards metastatic state. AGR2 protein was shown to regulate several cancer-associated processes including cellular proliferation, survival and drug resistance.

**Methods:**

The expression of AGR2 was analyzed in cancer cell lines exposed to TGF-β alone or to combined treatment with TGF-β and the Erk1/2 inhibitor PD98059 or the TGF-β receptor specific inhibitor SB431542. The impact of AGR2 silencing by specific siRNAs or CRISPR/Cas9 technology on EMT was investigated by western blot analysis, quantitative PCR, immunofluorescence analysis, real-time invasion assay and adhesion assay.

**Results:**

Induction of EMT was associated with decreased AGR2 along with changes in cellular morphology, actin reorganization, inhibition of E-cadherin and induction of the mesenchymal markers vimentin and N-cadherin in various cancer cell lines. Conversely, induction of AGR2 caused reversion of the mesenchymal phenotype back to the epithelial phenotype and re-acquisition of epithelial markers. Activated Smad and Erk signaling cascades were identified as mutually complementary pathways responsible for TGF-β-mediated inhibition of AGR2.

**Conclusion:**

Taken together our results highlight a crucial role for AGR2 in maintaining the epithelial phenotype by preventing the activation of key factors involved in the process of EMT.

**Electronic supplementary material:**

The online version of this article (doi:10.1186/s12885-017-3537-5) contains supplementary material, which is available to authorized users.

## Background

Epithelial-mesenchymal transition (EMT) is a reversible process of cellular reprogramming that plays an important role in normal development and injury response. In the last decades more interest has focused on the role of the oncogenic type of EMT in metastasis. Phenotypic changes occur during EMT, such as the loss of epithelial proteins including E-cadherin, cytokeratins and claudins, and the acquisition of mesenchymal proteins such as N-cadherin and vimentin [1]. Following these changes at the level of gene expression, polarized epithelial cells undergo morphological changes into spindle-shaped migratory mesenchymal cells [2]. Acquisition of a mesenchymal-like phenotype is usually associated with decreased cellular proliferation [3] and supports the escape of cells from the primary tumor site. However, phenotypic reversion to epithelial-like along with increased proliferation is essential for disseminated tumor cells to successfully colonize distant sites. The initiation and regulation of EMT is ensured by various growth and differentiation factors that orchestrate the activation of different signaling pathways and a wide range of transcription factors. Among these, transforming growth factor beta (TGF-β) has received much attention as a major inducer of EMT during embryogenesis, fibrosis and cancer progression [4, 5]. TGF-β-induced signaling may activate two independent pathways, Smad-dependent (transduction of signal by Smad proteins) and Smad-independent cascades including MAPK, PI3K/Akt/mTOR and Rho GTPase [6–10].

Human Anterior gradient 2 protein (AGR2) belongs to the family of Anterior gradient proteins, originally identified in Xenopus laevis as a potential secretory protein that is highly expressed in *Xenopus* eggs [11]. AGR2 is classified as a member of the protein disulfide isomerase family (PDI), a group of endoplasmic reticulum (ER)-resident proteins [12]. As a protein disulfide isomerase, AGR2 is thought to be involved in protein folding and maturation of client proteins (e.g. mucins MUC2, MUC5 and MUC1) and in maintaining ER homeostasis [13–17]. ER stress caused by accumulation of misfolded proteins may stimulate the unfolded protein response that in turn increases the expression of AGR2. Following its upregulation, AGR2 participates in the attenuation of degradation processes and prevents the induction of apoptosis, leading to increased cellular survival [12, 14, 18].

Alterations of AGR2 expression in cancer cells are reflected by the upregulation of cellular proliferation, tumor growth, inhibition of p53 and increased cellular survival, invasiveness and migration [19–21]. Proteins of AGR family were originally identified as estrogen receptor regulated [22, 23]. However, subsequent studies showed the contribution of other hormone-dependent as well as hormone-independent pathways in regulating AGR2 expression [24–26]. Pro-survival oncogenic pathways responsible for regulation of AGR2 expression along with the involvement of AGR2 in cellular adhesion and interaction with the extracellular matrix indicate the important function of AGR2 in the migration and invasiveness of cancer cells [27, 28]; however the precise mechanism remains to be elucidated.

To investigate the effect of TGF-β treatment on AGR2 expression, we used four different cancer cell lines expressing various levels of EMT markers in order to generalize the role of AGR2 in response to TGF-β treatment. Although these cells differed in classical EMT markers, AGR2 expression decreased in all tested cell lines in association with acquisition of a mesenchymal-like phenotype, as documented by changes in the levels of epithelial and mesenchymal markers. In contrast, increased expression of AGR2 was accompanied by an epithelial-like phenotype. Taken together, these data underscore the function of AGR2 in maintaining the epithelial phenotype and its role in re-establishing an epithelial phenotype during the development of metastasis.

## Methods

### Cell lines and reagents

#### Cell lines

A549 (CCL-185), H1299 (CRL-5803) (lung adenocarcinoma), BT-474 (HTB-20) and MCF-7 (HTB-22) (estrogen receptor-positive breast cancer), Panc1 (CRL-1469) (pancreatic adenocarcinoma) and HEK-293 (CRL-1573) (embryonic kidney epithelial cells) were obtained from ATCC and maintained in DMEM supplemented with 10% FBS, 1% pyruvate and L-glutamine at 37 °C in a humified atmosphere of 5% CO_2_. Unless otherwise stated, cells were grown to 70–80% confluence prior to treatment. TGF-β was added to a final concentration of 1 ng/ml for 24 h or as indicated. For inhibition of Erk1/2, cells were treated for 2 h with PD98059 prior to TGF-β treatment for the next 24 h.

Transfection was carried out using 2 μg of plasmid or 50 pmol of siRNA per million cells. To silence AGR2, cells were transiently transfected with siRNAs against AGR2 or untargeted siRNA as control (all from Dharmacon, ThermoFisher Scientific). The Flp-In™ System (Invitrogen) was used to generate H1299-LZ4 cells containing a single integrated Flp Recombination Target (FRT) site. The coding sequence of the human *AGR2* gene was stably inserted into this site using Flp recombinase mediated site-specific DNA recombination to give H1299-LZ4-AGR2 cell line. Plasmid pcDNA3-AGR2 was used to express AGR2 in transiently transfected cells.

#### Antibodies

Akt2 (5B5), p-AKT (S473; 736E11), p44/42 MAPK (Erk1/2; 137F5), p-p44/42 MAPK (T202/Y204, D13.14.4E), p-Smad2 (S465/467; 138D4), Smad4, SNAI2, ZEB1 (all Cell Signaling Technology); AGR2 (K-31, in-house); AGR2 (1C3, Abnova); vimentin (V9, Dako); E-cadherin (NCH38, Dako; HECD1, Abcam; Cell Signaling); N-cadherin (3B9, Invitrogen); β-actin (C4, Santa Cruz Biotech); Alexa Fluor 488 goat anti-mouse IgG, Alexa Fluor 532 goat anti-rabbit IgG (both Abcam); α-tubulin (AA13, Sigma); HRP-conjugated swine anti-rabbit and HRP-conjugated rabbit anti-mouse (both Dako).

#### Reagents

Human TGF-β1 (R&D Systems), protease and phosphatase inhibitors (Sigma-Aldrich), PD98059 (Cell Signaling Technology), SB431542 (Sigma). CytoPainter Phalloidin-iFluor 594 Reagent (Abcam) was used for phalloidin staining.

### CRISPR/Cas9

The oligonucleotide used to generate gRNAs for the human *AGR2* gene (AGAGATACCACAGTCAAACC) was designed using the CRISPR design web tool (crispr.mit.edu ). The GFP-scrambled sequence (AACAGTCGCGTTTGCGACTGG) used as a control was described earlier [29]. Oligonucleotides were cloned into the LentiCRISPR-v2 vector using Esp3I restriction cloning.

A549 cells (1 × 10^6^) were transfected with 2 μg of LentiCRISPR-v2_AGR2 and 2 μg of LentiCRISPR-v2_scrambled (control), respectively. Two days after transfection, puromycin (final concentration 0.5 μg/mL) was added to the cells and after 3–4 weeks the pool of resistant cells was seeded as single colonies in 96-well plates. After 2–3 weeks, the clones were tested for AGR2 expression using Western blot. Two clones, named A549 KOAGR2 G2 and A549 KOAGR2 G9 with undetectable expression of AGR2 were selected for further experiments. For verification and identification of the inserted mutation, the genomic DNA of these clones was isolated, the target region for Cas9 in the coding sequence of *AGR2* gene was amplified and sequenced (Additional file 1: Figure S1). Mutations causing frame shift were detected in both clones.

### Gene expression

Total RNA was isolated using TRIzol reagent (Invitrogen). The cDNA was synthesized by reverse transcriptase (Life Technologies). q-PCR was performed using SYBR Green MasterMix (Roche), TaqMan Universal PCR MasterMix was used for 18S rRNA (Life Technologies) representing a parallel endogenous control to GAPDH. All samples were analyzed in triplicates. The primer sequences are described in Additional file 2: Table S1.

### Immunofluorescence microscopy

Cells were fixed with 3% paraformaldehyde and permeabilized with 0.1% Triton X-100. Then the cells were blocked with 1% BSA diluted in PBS supplemented with 0.1% Tween (BSA-PBS-T) for 1 h and incubated overnight with primary antibody diluted in 1% BSA-PBS-T. Next day, coverslips were washed with PBS and probed for 1 h with Alexa Fluor 488 goat anti-mouse IgG or Alexa Fluor 532 goat anti-rabbit IgG (Abcam). After washing, cells were incubated with DAPI diluted in PBS (1:2500) for 5 min. Coverslips were mounted with Vectashield and images captured using an Olympus BX41 microscope. Images were analyzed with CellSens software (Olympus).

### Western blot analysis

Cell lines treated for the indicated time periods were subjected to SDS-PAGE and Western blot analysis. Prior to harvesting, cells were washed twice with cold phosphate-buffered saline (PBS) and scraped into NET lysis buffer (150 mM NaCl, 1% NP-40, 50 mM Tris pH 8.0, 50 mM NaF, 5 mM EDTA pH 8.0) supplemented with protease and phosphatase inhibitor cocktails according to the manufacturer’s instructions (Sigma). After SDS-PAGE, samples were transferred to nitrocellulose membranes which were blocked with 5% milk in PBS supplemented with 0.1% Tween (PBS-T) for 1 h at room temperature and then incubated overnight at 4 °C with primary antibodies diluted in 5% milk with 0.1% Tween. Next day, membranes were washed three times with PBS-T and probed with horseradish peroxidase-conjugated secondary antibodies (1:1000) for 1 h at room temperature. Chemiluminiscent signals were developed using ECL solution (0,5 M EDTA pH 8.0, 90 mM coumaric acid, 1 M luminol, 200 mM Tris-HCl pH 9.4, Na-perborate × 4H_2_0, 50 mM Na-acetate pH 5.0) and visualized with GeneTools software (Syngene). Either α-tubulin or β-actin was used as a loading control and protein expression was normalized in relation to these proteins.

### Invasion assay

Real-time cell analysis (RTCA) of invasion was performed on the xCELLigence Real-Time Cell Analyzer DP device (Roche) as described in the supplier’s instruction manual. Briefly, cells were added to the upper chamber of a two-chamber device separated by a porous membrane (the CIM-16 plate) through a solid matrix. Electrical impedance was displayed as a dimensionless parameter termed cell index reflecting the tumor cells’ invasive capacity. Prior to cell seeding, the surface of the upper chamber was covered with a monolayer of Matrigel. 30,000–50,000 cells/well suspended in serum-free culture medium were then seeded in the upper chamber. Cell indexes were measured every 15 min for up to 48 h with the RTCA software (version 1.2, Roche).

### Scattering assay, adhesion assay, cell-matrix adhesion assay

A549 and A549 KOAGR2 cells were allowed to grow as distinct colonies by seeding at 1000 cells/10-cm plate. The effect of AGR2 knockout on cell scattering was evaluated at day 5 and photographed under a phase contrast microscope. For adhesion assays, 25,000 cells were seeded per well on 96-well plates. Cells were left to adhere for 2 h at 37 °C and then adherent cells were fixed and stained. For cell-matrix adhesion assays, each well of 24-well plates was coated with Matrigel and left to solidify for 4 h at 37 °C. Then 50,000 cells per well were seeded. After incubation for 90 min, unbound cells were removed and adherent cells were fixed and stained with crystal violet. To quantify cell attachment, each well was solubilized with 2% SDS and absorbance was measured at 595 nm using an automated plate reader.

### Statistical analysis

One-way analysis of variance (one-way ANOVA) was used to determine differences between the means of studied samples. *P* < 0.05 was considered as significant.

## Results

### TGF-β downregulates the expression of AGR2

**Fig. 1:**
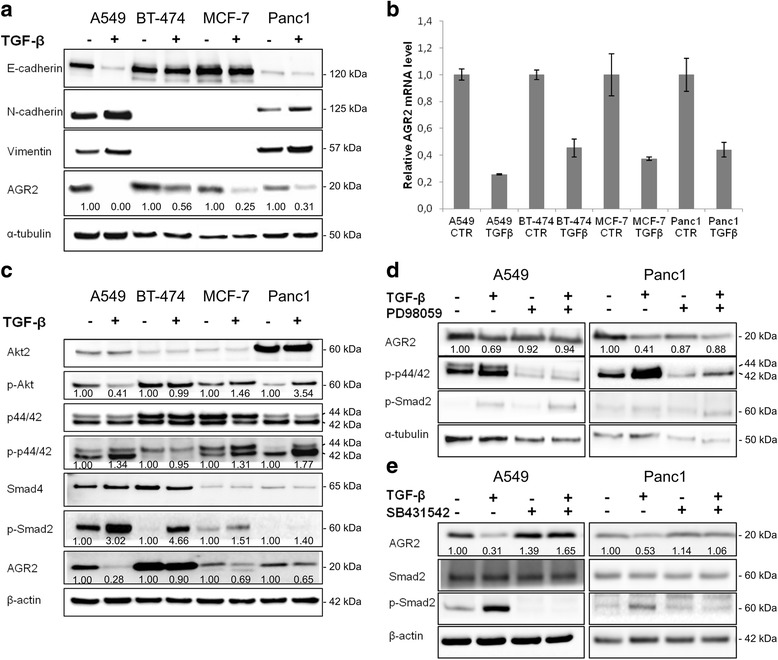
AGR2 expression is suppressed by TGF-β. **a** Four different cell lines A549, BT-474, MCF-7 and Panc1 were treated with TGF-β for 24 h. α-tubulin was used as a loading control and for normalization of protein levels calculated by densitometric analysis. Fold changes in protein level were calculated in relation to TGF-β untreated cells for each cell line independently. **b** mRNA levels of *AGR2* were analyzed by RT-qPCR, *GAPDH* mRNA served as an endogenous control for data normalization. Data for qPCR are the mean +/− standard deviation obtained from three independent experiments. The decrease of *AGR2* mRNA level in response to TGF-β in comparison with untreated cells was statistically significant (*P* < 0.05) for all analyzed cell lines. ***c*** Selected cell lines were treated with TGF-β and the cellular lysates were analyzed by Western blot with specific antibodies as indicated. β-actin was used as a loading control and for normalization to determine fold changes that were calculated in relation to TGF-β untreated cells for each cell line independently. **d** A549 and Panc1 cells were pre-treated with PD98059 for 2 h and then co-treated with TGF-β as indicated for 24 h. The changes in AGR2 level, phosphorylation of both p44/42 and Smad2 were analyzed by Western blot. α-tubulin was used as a loading control and for normalization of protein levels determined by densitometric analysis. Fold changes in protein level were calculated in relation to untreated cells for both cell lines independently. **e** A549 and Panc1 cells were pre-treated with SB431542 for 2 h and then co-treated with TGF-β as indicated for 24 h. The changes in AGR2 protein level were analyzed by Western blot. β-actin was used as a loading control and for normalization to calculate fold changes that were determined in relation to untreated cells for both cell lines independently

Overexpression of AGR2 was shown to be associated with tumorigenesis and metastasis development in many different types of malignancies [21, 30, 31]. Four AGR2-positive cancer cell lines showing different epithelial mesenchymal transition traits were selected to investigate the effect of TGF-β on EMT induction with respect to AGR2 expression. Changes in the level of E-cadherin, N-cadherin and vimentin representing the hallmarks of TGF-β-induced EMT were observed (Fig. 1a). TGF-β dependent induction of N-cadherin and vimentin was observed in A549 and Panc1 cells that produce these mesenchymal determinants under normal conditions. In contrast, the level of E-cadherin was decreased. AGR2 protein was decreased in response to TGF-β in all tested cell lines, but to a varying extent, suggesting that TGF-β-mediated decrease in AGR2 protein level represents a cell-context dependent process (Fig. 1a). To understand whether TGF-β mediates AGR2 downregulation at the level of transcription, the effect of TGF-β on *AGR2* mRNA levels was analyzed. RT-qPCR revealed decreased *AGR2* mRNA levels in all cell lines exposed to TGF-β, indicating that TGF-β suppresses *AGR2* transcription (Fig. 1b).

### Mechanism of TGF-β dependent AGR2 downregulation

To determine cell signaling pathways involved in TGF-β-dependent inhibition of AGR2, the canonical Smad-dependent pathway and non-canonical Smad-independent pathways PI3K/Akt and Erk1/2 were investigated. The level of the common-mediator Smad (Smad4), showed only non-significant differences in response to TGF-β treatment (Fig. 1c). Thus we focused on activation of the receptor-regulated Smads, more specifically Smad2. We found that all tested cell lines exposed to TGF-β showed induction of Smad2 phosphorylation, thereby confirming activation of Smad-dependent signaling in response to TGF-β (Fig. 1c).

Analysis of Smad-independent signaling pathways revealed that, except for BT-474 cells showing the most elevated level of phosphorylated Smad2 in response to TGF-β, downregulation of AGR2 by TGF-β treatment is associated with increased Erk1/2 (p44/42) phosphorylation. Moreover, TGF-β treatment induced Akt phosphorylation at Ser473 in MCF-7 and Panc1 cells, whilst Akt phosphorylation was decreased in A549 cells (Fig. 1c). These data indicate activation of non-Smad signaling pathways in these three cell lines during TGF-β mediated EMT. To understand the crosstalk between TGF-β-induced Smad-dependent and Smad-independent pathways in relation to AGR2 expression, Erk1/2 signaling pathway was inhibited by the specific inhibitor PD98059 in A549 and Panc1 cells, selected due to the most prominent Erk1/2 activation in response to TGF-β. We found that although TGF-β treatment reduced AGR2 expression and induced phosphorylation of Smad2, combined treatment with MAPK inhibitor did not impair TGF-β-induced Smad2 phosphorylation, but prevented the downregulation of AGR2 observed in cells exposed only to TGF-β (Fig. 1d). An explanation would be that treatment with PD98059 impairs translocation of activated Smad complexes to the nucleus, a rate-limiting step in TGF-β signal transduction. Although MAPK-specific inhibitors do not influence the total protein expression of Smad proteins, they may significantly block their nuclear translocation [32, 33]. Indeed, immunofluorescence staining of A549 cells revealed that TGF-β treated cells show strong nuclear staining for Smad2/Smad3, in comparison with control cells showing diffuse cytoplasmic staining for Smad2/3 proteins, similar to cells exposed simultaneously to both TGF-β and PD98059 (Additional file 3: Figure S2).

Incubation with the selective inhibitor of the transforming growth factor beta superfamily receptor type 1, SB431542, that specifically blocks TGF-β-mediated activation of Smad proteins, abolished the TGF-β-induced downregulation of AGR2 in A549 and Panc1 cells (Fig. 1e), confirming that the inhibitory effect of TGF-β on AGR2 expression is mediated through Smad signaling controlled by activated MAPKs.

### Altered AGR2 expression affects EMT

**Fig. 2:**
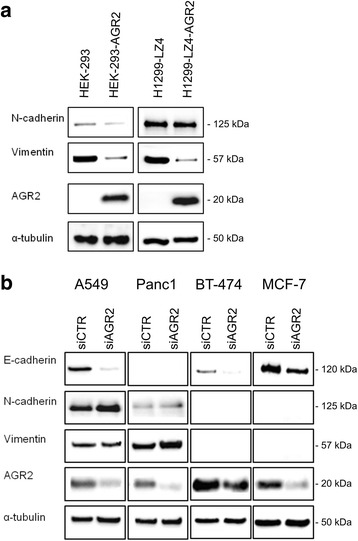
AGR2 expression regulates EMT. **a** Western blot analysis of HEK-293 cells either mock or AGR2 transiently transfected (*left part*) and H1299-LZ4 cells coupled with H1299-LZ4-AGR2 cells stably expressing AGR2 (*right part*). **b** Immunochemical analysis of A549, BT-474, MCF-7 and Panc1 cells transiently transfected with control (siCTR) or specific AGR2 siRNA (siAGR2). α-tubulin was used as a loading control, blots are representative of at least two experiments

To confirm that AGR2 represents a key component of TGF-β-induced EMT, we analyzed whether alteration in AGR2 expression may influence the EMT process independently of TGF-β. Two cell lines HEK-293 and H1299-LZ4 with immunochemically undetectable AGR2 expression were used. HEK-293 cells were transiently transfected with pcDNA3-AGR2 plasmid, while H1299-LZ4 cells were stably transfected with AGR2-coding sequences. Introduction of AGR2 to both cell lines decreased the levels of the mesenchymal markers vimentin and N-cadherin (Fig. 2a). E-cadherin was not detected in these cell lines. Moreover, immunofluorescence of H1299-LZ4-AGR2 cells showed rearrangement of the cytoskeleton, coupled with accumulation of vimentin in the perinuclear space, compared to the diffuse cytoplasmic localization of vimentin observed in AGR2-negative H1299 cells (Additional file 4: Figure S3A).

In contrast, cells endogenously expressing AGR2 transfected with AGR2 specific siRNA oligonucleotides showed decreased E-cadherin protein levels (Fig. 2b). In addition, downregulation of AGR2 in A549 and Panc1 caused an increase in N-cadherin and vimentin levels. Consistent with these data, immunofluorescence staining showed not only an increase in vimentin level but also its re-localization from the perinuclear space to the cytoplasm in response to AGR2 silencing (Additional file 4: Figure S3B). These results confirm AGR2 as a key element in EMT machinery, able to regulate changes in the EMT-associated phenotype independently of TGF-β treatment, since loss of AGR2 induced a shift from epithelial to mesenchymal phenotype and conversely, establishment of AGR2 expression initiated mesenchymal-epithelial transition (MET).

To map the cellular localization of the EMT related proteins, A549 cells were selected as representatives for immunofluorescence staining due to detectable expression of both epithelial (E-cadherin) and mesenchymal (vimentin) markers (Additional file 5: Figure S4A). Compared to untreated A549 cells, reduced AGR2 staining as well as a loss of E-cadherin was observed in response to TGF-β similarly to immunochemical analysis (Fig. 1a). AGR2 showed abundant localization at the perinuclear space in untreated cells, which reflects presence of this protein in endoplasmic reticulum as documented by co-localization with endoplasmic resident protein ERp57 (PDIA3, GRP58) (Additional file 5: Figure S4B). Similarly, vimentin staining was detected predominantly in the perinuclear space of untreated A549 cells, but TGF-β treatment caused diffuse cytoplasmic localization.

### AGR2 knockout mimics TGF-β-induced EMT in A549 cells

**Fig. 3:**
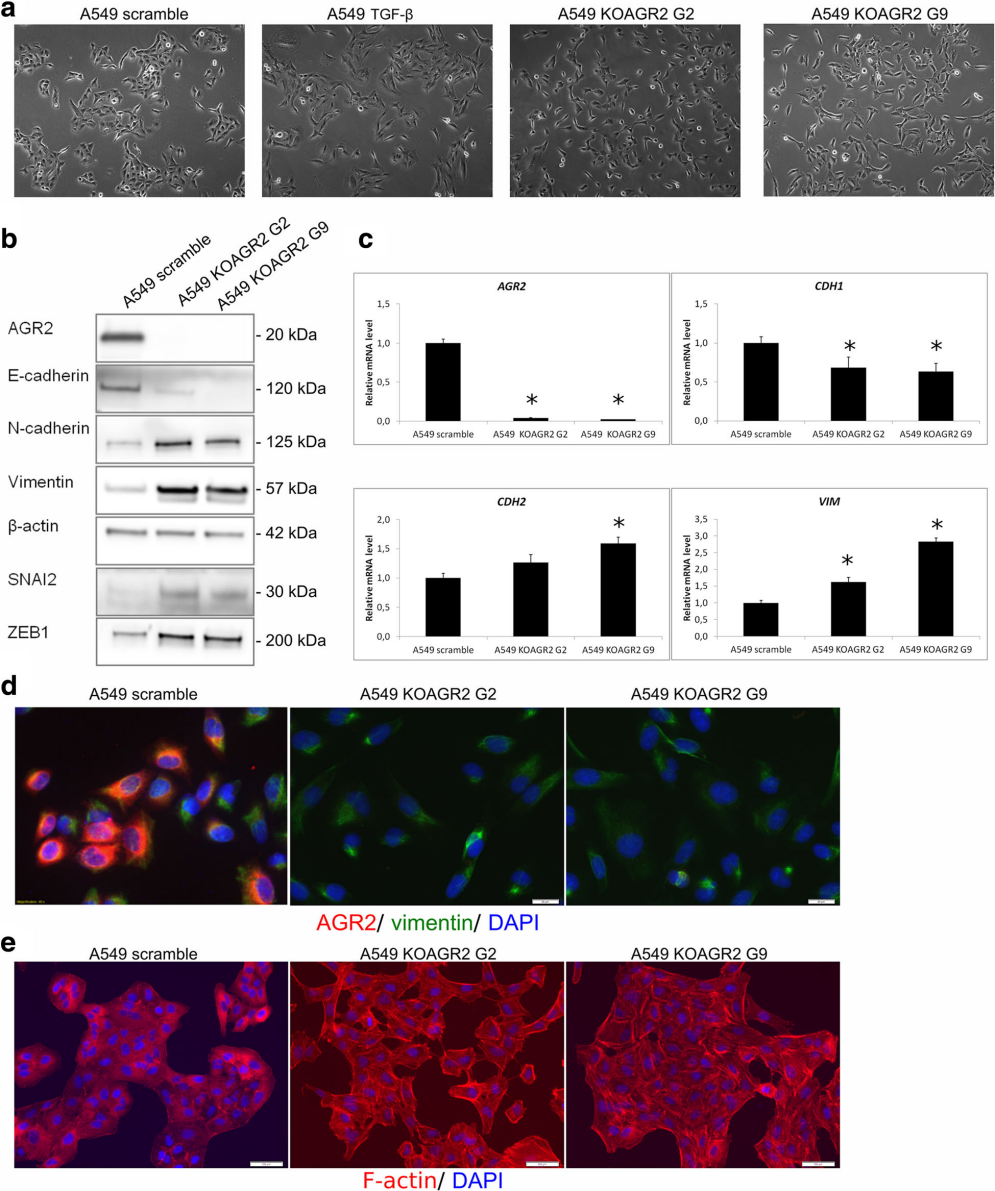
AGR2 loss mimics the changes associated with TGF-β-induced EMT. **a** A shift in morphology from rounded to spindle-like phenotype of cells was observed for both A549 KOAGR2 cells and A549 cells exposed to TGF-β in comparison with untreated A549 cells. **b** Protein levels of AGR2 and EMT markers were analyzed in protein lysates from A549 and two clones of A549 KOAGR2 cells named clone G2 and G9. **c** mRNA levels of *AGR2, CDH1, CDH2, VIM* were analyzed by RT-qPCR, *18S* rRNA served as an endogenous control for data normalization. Data for qPCR are the mean +/− standard deviation obtained from three independent experiments. Statistical significance (*P* < 0.05) is indicated by *asterisks*. **d** Changes in subcellular localization of vimentin in A549 and A549 KOAGR2 were analyzed using immunofluorescence. Staining for AGR2 (*red*), vimentin (*green*) and nucleic staining using DAPI was examined. **e** The A549 and A549 KOAGR2 were also stained with CytoPainter phalloidin-iFluor 594 reagent to detect morphological changes associated with F-actin reorganization. Nuclei were visualised with DAPI

CRISPR/Cas9 was used to generate A549 cells with AGR2 knockout (A549 KOAGR2). Interestingly, disruption of AGR2 expression in A549 cells led to the conversion from cobblestone-like shape (observed in control A549 cells) to a spindle-like mesenchymal phenotype, similar to the morphological changes observed in parental A549 cells in response to TGF-β treatment (Fig. 3a). Comparison between original A549 cells and A549 cells without AGR2 expression by Western blot and RT-qPCR revealed a decrease of E-cadherin and induction of vimentin and N-cadherin (Fig. 3b, c) in A549 KOAGR2 cells, similar to the observations made in response to transient AGR2 silencing (Fig. 2). Interestingly, the loss of AGR2 resulted in the induction of two EMT-positive regulators ZEB1 and SNAI2 (Fig. 3b), indicating that the presence of AGR2 is associated with decreased expression of these two EMT-inducing transcription factors. Immunofluorescence analysis confirmed that loss of AGR2 expression in A549 KOAGR2 cells is associated with the cellular re-localization of vimentin to the cytoplasm corresponding with the changes in cellular morphology, whilst in parental A549 cells vimentin seems to be co-localized with AGR2 in the perinuclear space (Fig. 3d). In addition, phalloidin staining showed that F-actin was mainly distributed in cell-cell borders and very few stress fibers were evident in control A549 cells, while A549 KOAGR2 cells demonstrated dramatic changes in the organization of F-actin, which was shifted from cell-cell contacts and formed stress fibers throughout the cytoplasm (Fig. 3e).

### AGR2 influences cellular adhesion and invasiveness

**Fig. 4:**
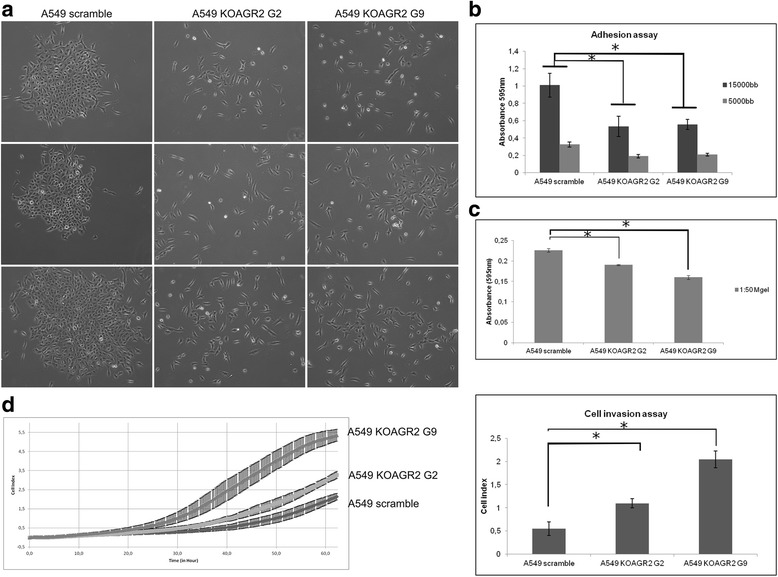
Knockout of AGR2 alters the cell-cell and cell-matrix adhesion and increases the invasiveness of A549 cells*.* **a** Control A549 cells show epithelial-like colonies with high level of cell-cell adhesion; both AGR2 knockout clones show reduced cell-cell adhesion and have spindle-shape morphology. Phase-contrast microscopic images were taken at day 5 after cell seeding. Single cell suspensions were placed on **b** uncoated or **c** Matrigel-coated tissue culture plates for 2 h for adhesion assay and for 90 min for cell-matrix adhesion assay. The *error bars* represent the standard deviation of the mean determined from three independent experiments, each performed in duplicate. **d** Cells were plated in the upper chamber of a CIM-16 plate coated with Matrigel. Real-time invasion of cells was measured on an Xcelligence Real-Time Cell Analyzer DP analyzer. Representative graph (*left*) for A549 and both clones of A549 KOAGR2 (G2, G9) shows invasiveness of A549 cells in relation to AGR2 expression. Each clone was determined in triplicate. Resulting graph (*right*) obtained from two independent experiments (representative experiment is shown on the *left*) shows cellular invasiveness determined at 60 h. Data are presented as the mean +/− standard deviation (*n* = 5). Statistical significance (*P* < 0.05) is indicated by *asterisks*

Functional changes frequently observed in EMT involve decreased cell-ECM adhesion and loss of cell-cell junctions representing important prerequisites for release of malignant cells from a primary tumor site. To determine the function of AGR2 in cell-cell contacts, we investigated the role of AGR2 in formation of cell-cell junctions. A549 scramble and A549 cells with AGR2 knockout were analyzed by a colony scattering assay measuring the ability of epithelial tumor cells to detach from colonies in culture, mimicking some aspects of tumor invasiveness. Cells were plated at a very low density (1000 cells/10-cm plate) and the morphology of colonies was analyzed 5 days after plating. Light microscopy images revealed that scramble A549 cells grew as tight clusters composed of cells with typical cobblestone-like epithelial morphology. In contrast, A549 KOAGR2 cells exhibited a more scattered distribution and elongated shape, a distinctive feature of mesenchymal-like cells forming poorly organized junctions between adjacent cells (Fig. 4a). In line with these observations we also found that cells with blocked AGR2 expression show lower cellular adhesion (Fig. 4b). Similarly, the cell-ECM substrate adhesion assays showed that AGR2 knockdown leads to a decreased ability of cells to adhere to Matrigel (Fig. 4c).

Cellular migration and invasiveness are two processes tightly associated with EMT. We found that loss of AGR2 does not alter migration (data not shown). However, invasion assays showed that AGR2 silencing increases the number of cells that invade through Matrigel-coated inserts (Fig. 4d). Taken together, these data confirm a protecting role of AGR2 in maintaining of epithelial cellular phenotype.

## Discussion

Epithelial-mesenchymal transition is widely accepted as the essential step in the initiation of metastasis development and cancer cell dissemination from primary tumors [3, 34]. However, to accomplish metastatic growth, mesenchymal migratory cells have to be reprogrammed back to obtain the original epithelial phenotype. The reversion occurs during the process known as mesenchymal-epithelial transition, representing a second crucial step in carcinogenesis that mediates tissue colonization and the development of metastatic tumors at secondary sites [35]. Despite considerable progress in research focused on EMT/MET crosstalk, the whole process is still not fully understood.

In recent years, the role of AGR2 in tumor development and progression is becoming more and more intensively studied [13, 36]. The contribution of AGR2 to malignant transformation [21], drug resistance [19, 37, 38] and metastasis development [30, 31] have already been reported, however the mechanism of action, as well as the scope of AGR2 functions remain unclear. In the present study, we identified AGR2 as an important factor contributing to the maintenance of the epithelial phenotype of tumor cells.

We found that AGR2 expression positively correlates with the expression of the epithelial marker E-cadherin, while TGF-β-induced reduction of AGR2 was concomitant with the classical features of mesenchymal cells such as the loss of E-cadherin, induction of N-cadherin and morphological changes arising from cytoskeleton reorganisation including diffuse cytoplasmic distribution of vimentin and re-localization of actin. These data demonstrate an important role of AGR2 in maintaining epithelial phenotypes. Interestingly, transient AGR2 silencing as well as stable knockout mimicked the initiation of EMT. Accordingly, exogenous expression of AGR2 in two different cellular models confirmed the capacity of AGR2 to revert a mesenchymal phenotype to an epithelial phenotype, indicating not only the important role of AGR2 expression in EMT, but also that elevated AGR2 expression may prevent the acquisition of a mesenchymal phenotype by cancer cells. A key role of AGR2 in EMT is also supported by the similar morphological changes in A549 cells exposed to TGF-β or with silenced AGR2, showing a shift from a classical cobblestone epithelial morphology to a fibroblast-like morphology and F-actin rearrangement. These findings representing a hallmark of epithelial cancer metastasis development are also consistent with our experiments confirming involvement of AGR2 in preventing cellular invasiveness and maintaining cell-cell junctions along with adhesion to ECM. Interestingly, reduced cell adhesion was also observed after abrogation of AGR-2 expression in prostate cancer cells [39].

The validation of TGF-β as a potent suppressor of AGR2 expression in our panel of tumor cell lines is consistent with the previous findings that have characterised *AGR2* as a TGF-β responsive gene [15]. The important role of Smad4 in triggering AGR2 downregulation in human pancreatic cancer cells exposed to TGF-β has recently been shown [15]. Subsequently, AGR2 downregulation was determined by immunohistochemical staining and correlated with vimentin expression and reduced expression of membranous E-cadherin in pancreatic precursor neoplastic lesions, as well as pancreatic ductal adenocarcinomas [40]. Additionally, our data indicate that TGF-β suppressive function on AGR2 expression is not exclusively dependent on the Smad-canonical pathways. Some previous reports suggested that MAPK signaling positively regulates AGR2 expression [26], however TGF-β-induced MAPK activation in our cell lines led to AGR2 downregulation. Consistent with recent literature [26, 41], we confirm the role of Erk1/2 signaling pathway in regulating AGR2 by showing that inhibition of Erk1/2 reduces AGR2 protein levels. Interestingly, although individual treatment with either Erk1/2 inhibitor or TGF-β reduces AGR2 expression, their combination results in negligible changes in AGR2 protein levels (Fig. 1). To elucidate this phenomenon, we found that inhibition of the Erk1/2 pathway in A549 cells does not affect activation of Smad signaling in response to TGF-β but rather impairs nuclear import of the Smad complex. The blockade of nuclear translocation may in turn prevent the suppressive effect of TGF-β/Smad signaling on AGR2 expression. In accordance, previously published data showed that treatment with the MAPK signaling inhibitor, PD98059, blocked the formation of Smad complex [42] and impaired binding of Smad complexes to Smad-responsive elements [43, 44]. Thus, we suggest that TGF-β dependent induction of the MAPK signaling cascade is essential for the transcriptional activity of the Smad-dependent pathway that results in TGF-β mediated AGR2 downregulation.

In addition to identifying the mechanism responsible for TGF-β-dependent repression of AGR2, we also revealed that AGR2 actively interferes with the EMT process to maintain an epithelial phenotype via inhibition of EMT-inducing transcription factors ZEB1 and SNAI2. Another potential mechanism by which AGR2 contributes to blocking EMT is the inhibitory effect on p38 MAPK signaling, which is frequently involved in triggering EMT [19, 45].

Very recently, several studies showed that extracellular AGR2 promotes epithelial morphogenesis and tumorigenesis by interruption of adherens junctions, disruption of basal laminin and activation of fibroblast-associated cancer invasion [37, 46]. That elevated levels of secreted AGR2 mediate more aggressive cellular phenotypes associated with loss of cellular polarity, remodeling of extracellular matrix and increased invasiveness may appear to be inconsistent with our results. However, it should be noted that our results describe the role of intracellular AGR2, which protects the epithelial cellular phenotype by preventing EMT induction, in accordance with other reports focused on the function of intracellular AGR2 [39, 47]. Taken together, the role of AGR2 in EMT is dependent on its localization, which seems to be responsible for a set of different impacts on cancer cell migration, invasiveness and metastasis development. However, the precise mechanism(s) regulating the levels of intracellular and extracellular AGR2 and/or their balance remain to be further investigated. Proteins with opposite functions depending on their presence inside or outside cells were already described in detail, for example heat shock proteins, namely Hsp70, or calcium-binding proteins S100A8 and S100A9 [48–50]. Moreover, the well-studied keeper of epithelial phenotype E-cadherin showed pro-metastatic function when cleaved and localized in the extracellular space [51].

## Conclusions

In summary, our findings provide new insights into the regulation of AGR2 as well as AGR2 functions with respect to TGF-β signaling, clarifying the role of AGR2 in EMT/MET. Our data confirm AGR2 as a keeper of epithelial phenotypes, showing a positive role of AGR2 in the regulation of the epithelial marker E-cadherin and a negative effect on the mesenchymal markers vimentin and N-cadherin. In primary tumors, AGR2 is predominately responsible for increased proliferation and growth of malignant cells, but later during the metastatic cascade AGR2 contributes to successful settling and adhesion of disseminated cells in secondary sites, their adaptation to the tumor milieu and stimulation of secondary tumor growth. In relation to molecular changes associated with EMT and MET phenotypes, AGR2 appears to be a key regulator of these processes indicating a dual role of AGR2 in cancer.

## Additional files

Additional file 1: Figure S1. Sequencing analysis to identify mutations prepared by CRISPR/Cas9 that are responsible for AGR2 gene knockout. (PDF 345 kb)

Additional file 2: Table S1. Sequences of the primers used in quantitative PCR analysis. (PDF 213 kb)

Additional file 3: Figure S2. The effect of PD98059 inhibitor on TGF-β induced nuclear accumulation of Smad2/Smad3 complex. (PDF 352 kb)

Additional file 4: Figure S3. The effect of AGR2 expression on vimentin cellular localization. A scale bars correspond to 20 μm. (PDF 319 kb)

Additional file 5: Figure S4. The effect of TGF-β treatment on protein levels and cellular localization of selected EMT markers. A scale bars correspond to 20 μm. (PDF 313 kb)

## Abbreviations

AGR2: Anterior gradient protein 2
EMT: Epithelial-mesenchymal transition
ER: Endoplasmic reticulum
MET: Mesenchymal-epithelial transition
PDI: Protein disulfide isomerase
TGF-β: Transforming growth factor beta
