# Supplementary material for: Suppression of AGR2 in a TGF-β-induced Smad regulatory pathway mediates epithelial-mesenchymal transition
Source: BMC Cancer. 2017 Aug 15;17:546. doi: 10.1186/s12885-017-3537-5 (PMC5557473; doi:10.1186/s12885-017-3537-5)
Supplement: Supplementary file 1 — Sequencing analysis to identify mutations prepared by CRISPR/Cas9 that are responsible for AGR2 gene knockout. (PDF 345 kb) [file 12885_2017_3537_MOESM1_ESM.pdf]

A

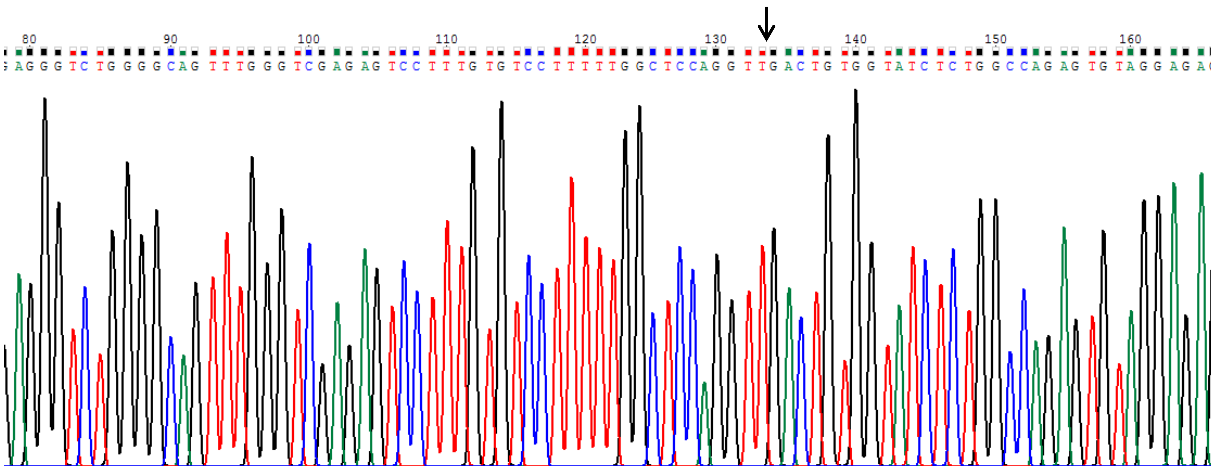

Score	Expect	Identities	Gaps	Strand	Frame
254 bits(137)	2e-71()	140/141(99%)	1/141(0%)	Plus/Minus	

Features:

```
Query  69   ACCTCTGGAGAGGGTCTGGGGCAGTTTGGGTCGAGAGTCCTTTGTGTCCTTTTTGGCTCC  128
Sbjct  141  ............................................................  82

Query  129  AGGTT-GACTGTGGTATCTCTGGCCAGAGTGTAGGAGAGGGCCACAAGGAGCAAGAATGC  187
Sbjct  81   .....T......................................................  22

Query  188  TGACACTGGAATTTTCTCCAT  208
Sbjct  21   .....................  1
```

B

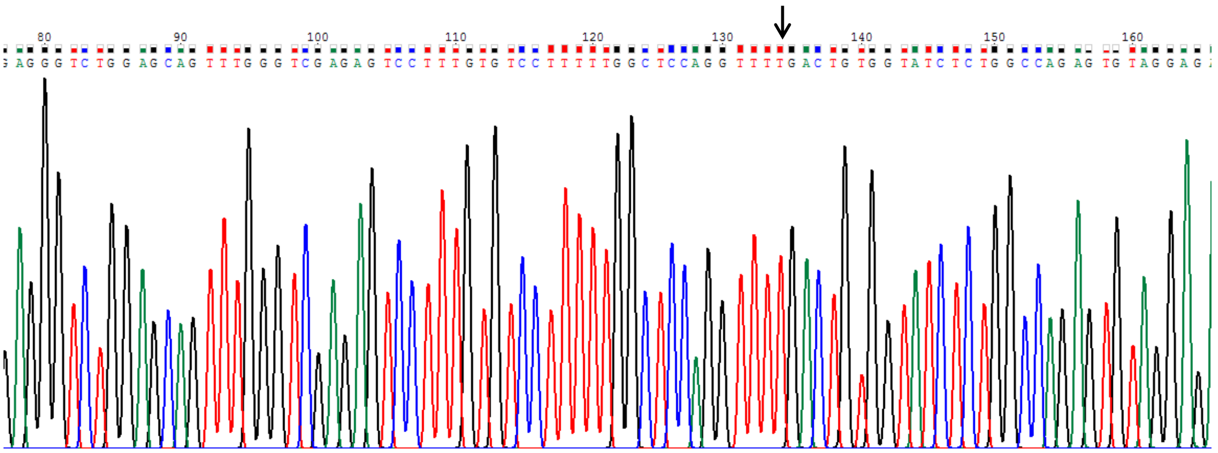

Score	Expect	Identities	Gaps	Strand	Frame
250 bits(135)	3e-70()	140/142(99%)	1/142(0%)	Plus/Minus	

Features:

```
Query  68   ACCTCTGGAGAGGGTCTGGAGCAGTTTGGGTCGAGAGTCCTTTGTGTCCTTTTTGGCTCC  127
Sbjct  141  ...................G........................................  82

Query  128  AGGTTTTGACTGTGGTATCTCTGGCCAGAGTGTAGGAGAGGGCCACAAGGAGCAAGAATG  187
Sbjct  81   ......-.....................................................  23

Query  188  CTGACACTGGAATTTTCTCCAT  209
Sbjct  22   ......................  1
```

C

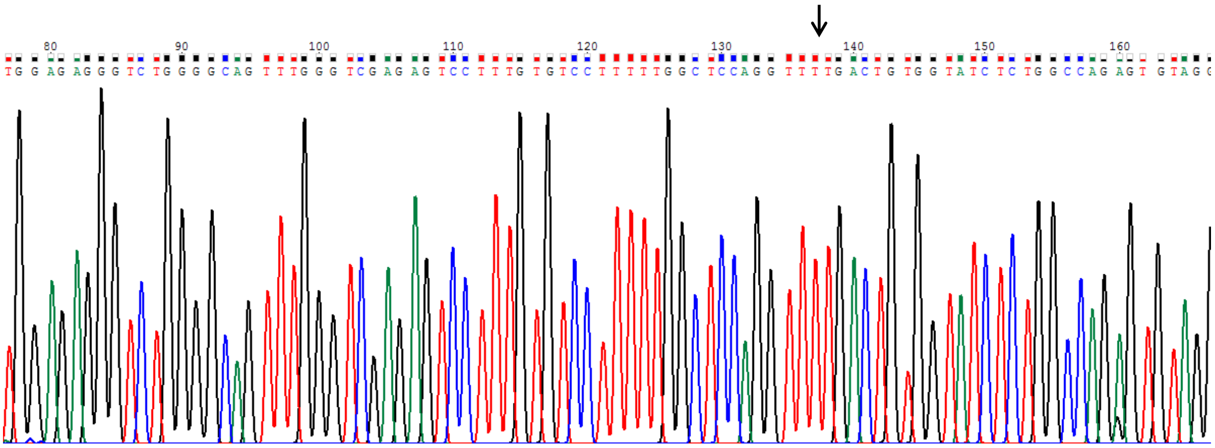

Score	Expect	Identities	Gaps	Strand	Frame
255 bits(138)	6e-72()	141/142(99%)	1/142(0%)	Plus/Minus	

Features:

```
Query  72   ACCTCTGGAGAGGGTCTGGGGCAGTTTGGGTCGAGAGTCCTTTGTGTCCTTTTTGGCTCC  131
Sbjct  141  ............................................................  82

Query  132  AGGTTTTGACTGTGGTATCTCTGGCCAGAGTGTAGGAGAGGGCCACAAGGAGCAAGAATG  191
Sbjct  81   ......-.....................................................  23

Query  192  CTGACACTGGAATTTTCTCCAT  213
Sbjct  22   ......................  1
```

Figure S1: **Sequencing analysis to identify mutations prepared by CRISPR/Cas9 that are responsible for *AGR2* gene knockout.**

(A) Deletion 76T was observed in A549 AGR2KO clone G9; **(B)** Insertion 75T was detected in both A549 AGR2KO clones G9 and G2; **(C)** Insertion 75T was also detected in the second allele of A549 AGR2KO G2 clone.
