# Supplementary material for: Suppression of AGR2 in a TGF-β-induced Smad regulatory pathway mediates epithelial-mesenchymal transition
Source: BMC Cancer. 2017 Aug 15;17:546. doi: 10.1186/s12885-017-3537-5 (PMC5557473; doi:10.1186/s12885-017-3537-5)
Supplement: Supplementary file 2 — Sequences of the primers used in quantitative PCR analysis. (PDF 213 kb) [file 12885_2017_3537_MOESM2_ESM.pdf]

Gene	Forward	Reverse
AGR2	**GGAGCTCTATATAAATCCAAGACAAGCA**	**GCCAATTTCTGGATTTCTTTATTTTC**
GAPDH	**GAAGGTGAAGGTCGGAGTC**	**GAAGATGGTGATGGGATTTC**
CDH1	**GACACACCCCCTGTTGGTGT**	**CAGCCATCCTGTTTCTCTTTCAA**
CDH2	**ATCTCGGGTCAGCTGTCGG**	**GGCTATCTGCTCGCGATCC**
VIM	**GGAACAGCATGTCCAAATCGA**	**GCCGTGAGGTCAGGCTTG**
ZEB1	**CACTGGTGGTGGCCCATTAC**	**TGCACCATGCCCTGAGG**
SNAI2	**TGTGTGGACTACCGCTGCTC**	**GAGAGGCCATTGGGTAGCTG**

Table S1: Sequences of the primers used in quantitative PCR analysis.
