# Supplementary material for: Suppression of AGR2 in a TGF-β-induced Smad regulatory pathway mediates epithelial-mesenchymal transition
Source: BMC Cancer. 2017 Aug 15;17:546. doi: 10.1186/s12885-017-3537-5 (PMC5557473; doi:10.1186/s12885-017-3537-5)
Supplement: Supplementary file 3 — The effect of PD98059 inhibitor on TGF-β induced nuclear accumulation of Smad2/Smad3 complex. (PDF 352 kb) [file 12885_2017_3537_MOESM3_ESM.pdf]

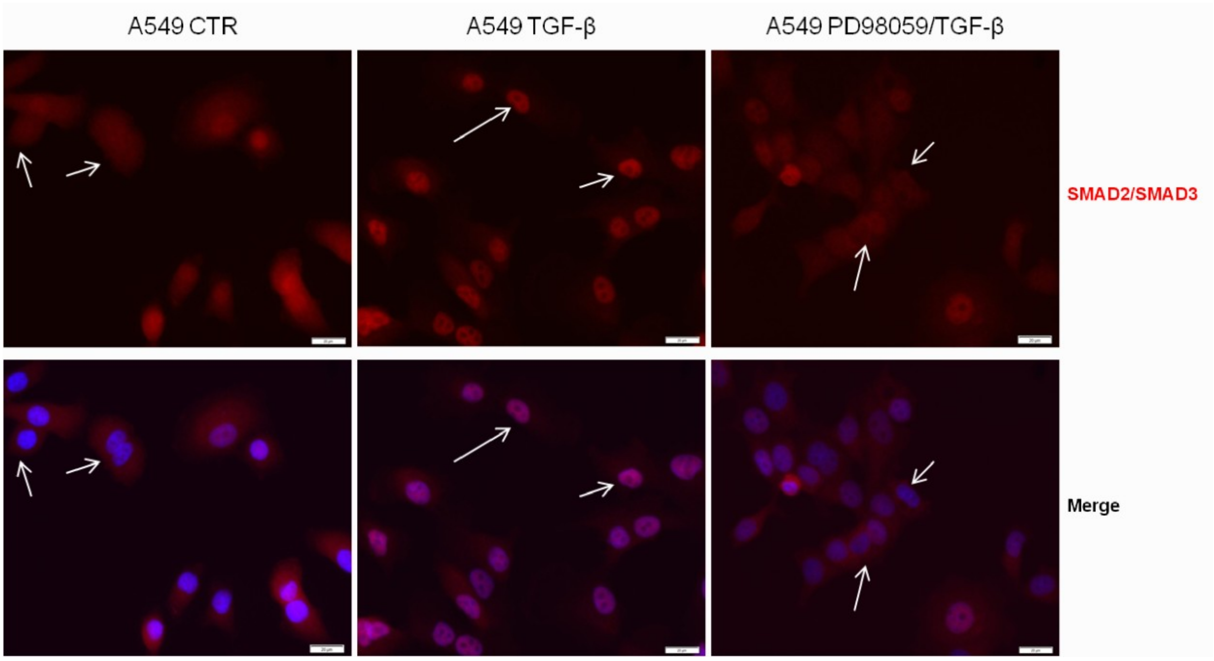

Figure S2: **The effect of PD98059 inhibitor on TGF-β induced nuclear accumulation of Smad2/Smad3 complex.**

The subcellular localization of Smad proteins was determined in A549 cells pre-treated for 2 h with 25 nM PD98059 and then exposed to 1 ng/ml of TGF-β for 24 h. Fluorescent microscopy was used to analyze cells, the nuclei were visualized by DAPI staining. A scale bars correspond to 20 μm. Arrows point to the representative cells highlighting the difference in subcellular localization.
