# Supplementary material for: Suppression of AGR2 in a TGF-β-induced Smad regulatory pathway mediates epithelial-mesenchymal transition
Source: BMC Cancer. 2017 Aug 15;17:546. doi: 10.1186/s12885-017-3537-5 (PMC5557473; doi:10.1186/s12885-017-3537-5)
Supplement: Supplementary file 4 — The effect of AGR2 expression on vimentin cellular localization. A scale bars correspond to 20 μm. (PDF 319 kb) [file 12885_2017_3537_MOESM4_ESM.pdf]

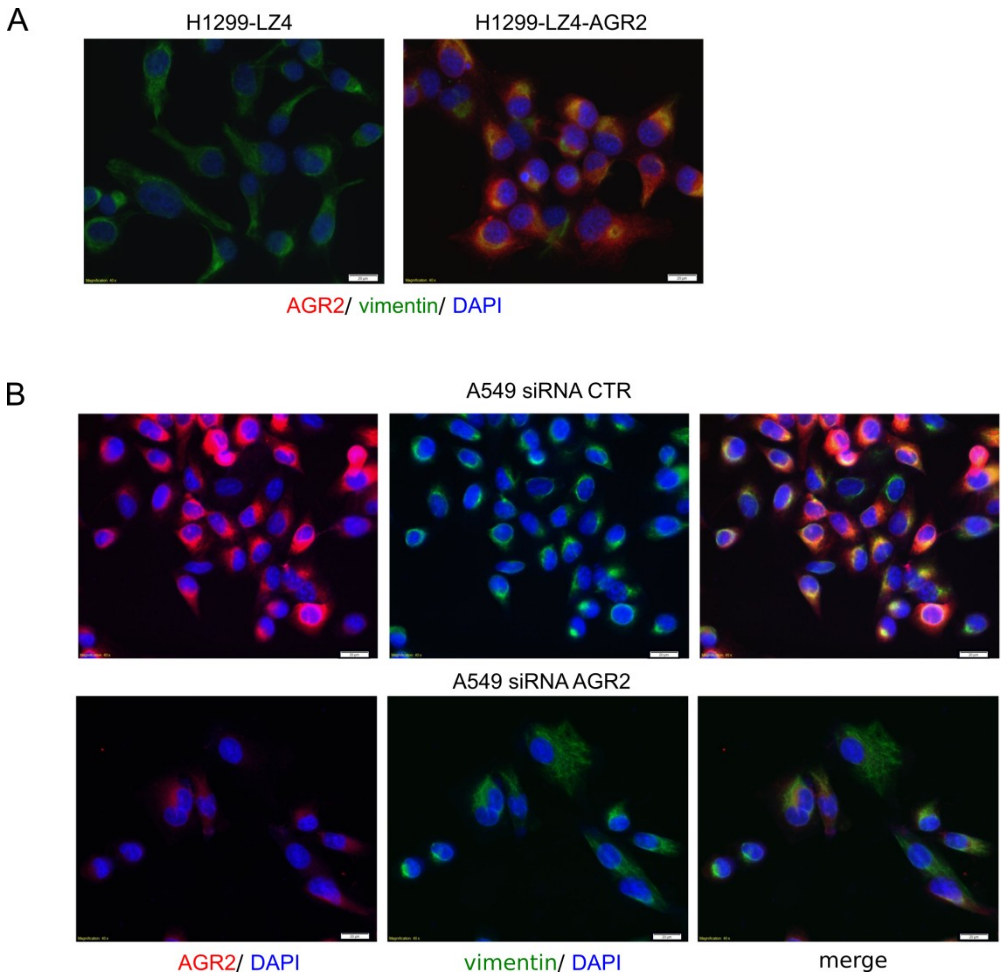

Figure S3: **The effect of AGR2 expression on vimentin cellular localization. A scale bars correspond to 20 µm.**

(A) Changes in the expression of AGR2 and the localization of mesenchymal marker vimentin were visualized by the immunofluorescence staining in H1299-LZ4 and H1299-LZ4-AGR2 cells. The nuclei were stained with DAPI. **(B)** A549 cells were transiently transfected with AGR2 specific siRNA or with the control siRNA, 48 h later the cells were fixed, permeabilized and immunostained with anti-AGR2 and anti-vimentin antibodies. Nuclei were visualized with DAPI.
