# Supplementary material for: Suppression of AGR2 in a TGF-β-induced Smad regulatory pathway mediates epithelial-mesenchymal transition
Source: BMC Cancer. 2017 Aug 15;17:546. doi: 10.1186/s12885-017-3537-5 (PMC5557473; doi:10.1186/s12885-017-3537-5)
Supplement: Supplementary file 5 — The effect of TGF-β treatment on protein levels and cellular localization of selected EMT markers. A scale bars correspond to 20 μm. (PDF 313 kb) [file 12885_2017_3537_MOESM5_ESM.pdf]

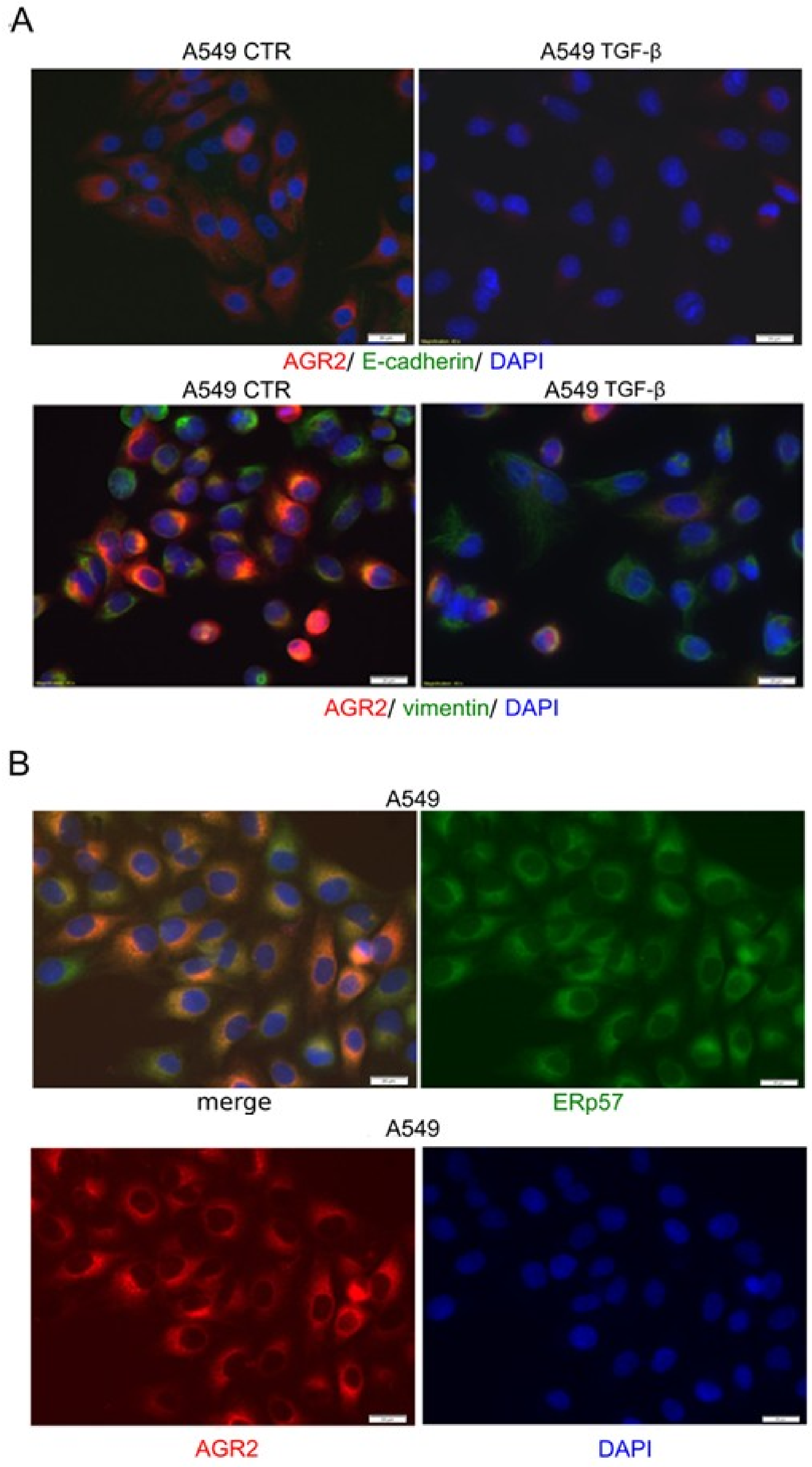

Figure S4: **The effect of TGF-β treatment on protein levels and cellular localization of selected EMT markers. A scale bars correspond to 20 µm.**

(A) TGF-β induced changes in the subcellular localization were determined in A549 cells exposed to TGF-β for 24 h. **(B)** Co-localization of AGR2 and ERp57. The nuclei were visualized by DAPI.
